# The importance of plant-derived biomaterials for cardiac tissue engineering

**DOI:** 10.31744/einstein_journal/2021CE6678

**Published:** 2021-06-28

**Authors:** Igor Carreiro Ramalho, Carlos Magno da Costa Maranduba, Leandro Marques de Resende, Pâmela de Souza Lourenço

**Affiliations:** 1 Universidade Federal de Juiz de Fora Juiz de ForaMG Brazil Universidade Federal de Juiz de Fora, Juiz de Fora, MG, Brazil.; 2 Centro Universitário Geraldo Di Biase Barra do PiraíRJ Brazil Centro Universitário Geraldo Di Biase, Barra do Piraí, RJ, Brazil.

Dear Editor,

The paper titled “Cardiac tissue engineering: current state-of-the-art materials, cells and tissue formation”,^(1)^ published in this journal contributed with the discussion on the role of tissue engineering. Challenges from the area involve reaching desirable biological and physical proprieties in biomaterials, such as structures that promote a high efficiency in the delivery of nutrients^(2-4)^ by supplying the lack of afunctional vascular network- one of the main factors that affect the clinical translation of tissue engineering.

Microvasculature cannot yet be effectively reproduced by current techniques of biofabrication.^(4)^ Biomaterials originated from decellularized tissues of animals are alternatives to this problem/issue, however, they have higher cost to be obtained, their availability is limited and, sometimes, they present little compatibility, and low durability.^(3)^ Plant-derived biomaterials constitute an alternative that present limited degradation by beingresistant to enzymatic action. In addition, they have long service life,^(2)^ low cost and greater availability.^(4)^ Plant-derived biomaterials have high surface area, interconnected porosity, and preexisting vascular networks.^(2,3)^ Studies on celluloses have also shown its application in wound healing.^(5)^

In 2017 Gershlak et al.,^(4)^demonstrated that human mesenchymal stem cell-derived cardiomyocytes had the ability to manipulate calcium and spontaneous contractile function after recellularization in biomaterials obtained from spinach leaves. In addition to maintain the vascular and topographic features, the decellularized leaves were able to support the flow of particles within the size of red blood cells.^(4)^ Given that several synthetic biomaterials derive from non-renewal resources, they may still generate toxic sub-products, and due to the major concern with the environment, decellularized plants used as biomaterialscan represent a “green” technology that is easily accessible, beingextremely relevant in our current scenario.
