# Supplementary material for: The importance of plant-derived biomaterials for cardiac tissue engineering
Source: Einstein (Sao Paulo). 2021 Jun 28;19:eCE6678. doi: 10.31744/einstein_journal/2021CE6678 (PMC8244926; doi:10.31744/einstein_journal/2021CE6678)
Supplement: Translation

# Translation: A importância dos biomateriais derivados de plantas para a engenharia de tecidos cardíacos

**Authors:** Igor Carreiro Ramalho, Carlos Magno da Costa Maranduba, Leandro Marques de Resende, Pâmela de Souza Lourenço

**Affiliations:** 1 Brasil Universidade Federal de Juiz de Fora, Juiz de Fora, MG, Brasil.; 2 Brasil Centro Universitário Geraldo Di Biase, Barra do Piraí, RJ, Brasil.

Caro Editor,

O artigo “Engenharia de tecidos cardíacos: atual estado da arte a respeito de materiais, células e formação tecidual”,^(1)^ publicado nesta revista, contribui com a discussão sobre o papel da engenharia de tecidos. Desafios nessa área envolvem alcançar propriedades biológicas e físicas desejáveis em biomateriais, como estruturas que favoreçam uma alta eficiência na entrega de nutrientes,^(2-4)^ suprindo a falta de uma rede vascular funcional – um dos principais fatores que afetam a tradução clínica da engenharia de tecidos.

A microvasculatura ainda não pode ser reproduzida com eficácia pelas técnicas atuais de biofabricação.^(4)^ Biomateriais originados de tecidos descelularizados de animais são uma alternativa a essa problemática, porém eles possuem alto custo de obtenção, disponibilidade limitada e, muitas vezes, baixa biocompatibilidade, além de pouca durabilidade.^(3)^ Alternativamente, biomateriais derivados de plantas possuem degradação limitada, sendo resistentes à ação enzimática e tendo maior vida útil,^(2)^baixo custo e maior disponibilidade.^(4)^ Ainda, possuem alta área de superfície, porosidade interconectada e redes vasculares preexistentes.^(2,3)^ Além disso, estudos com celulose mostram sua aplicação na cicatrização de feridas.^(5)^

Em 2017, Gershlak et al.,^(4)^ mostraram que cardiomiócitos derivados de células-tronco mesenquimais humanas apresentaram capacidade de manipulação de cálcio e função contrátil espontânea, após a recelularização em biomateriais obtidos de folhas de espinafre. As folhas descelularizadas, além de manterem as características vasculares e topográficas, foram capazes de suportar o fluxo de líquidos e de células do tamanho de um glóbulo vermelho.^(4)^ Como muitos biomateriais sintéticos são derivados de recursos não renováveis, podendo ainda gerar subprodutos tóxicos, e devido à preocupação geral com o meio ambiente, as plantas descelularizadas utilizadas como biomateriais poderiam representar uma tecnologia “verde” de fácil acesso, sendo extremamente relevantes em nosso cenário atual.
